# Supplementary material for: From sequence to enzyme mechanism using multi-label machine learning
Source: BMC Bioinformatics. 2014 May 19;15:150. doi: 10.1186/1471-2105-15-150 (PMC4229970; doi:10.1186/1471-2105-15-150)
Supplement: Additional file 2 — Java code of ml2db. Additional file ml2db_code.tar.gz contains the Java source code to run the multi-label machine learning experiments and save the results to database. The code’s Javadoc is included. [file 1471-2105-15-150-S2.zip › additional file 2/ml2db/ml2db/doc/mulan/data/Stratification.html]

Stratification


JavaScript is disabled on your browser.


- Overview
- Package
- Class
- Use
- Tree
- Deprecated
- Index
- Help

- Prev Class
- Next Class

- Frames
- No Frames

- All Classes

- Summary:
- Nested |
- Field |
- Constr |
- Method

- Detail:
- Field |
- Constr |
- Method


mulan.data

## Interface Stratification

- All Known Implementing Classes:
  :   IterativeStratification, LabelPowersetStratification

  ---

    

  ```
  public interface Stratification
  ```

  Interface for multi-label stratification methods

  Version:
  :   2012.05.08

  Author:
  :   Grigorios Tsoumakas

- - ### Method Summary

    Methods

    | Modifier and Type | Method and Description |
    | --- | --- |
    | `MultiLabelInstances[]` | `stratify(MultiLabelInstances data, int folds)` Creates a number of folds via stratified sampling |

- - ### Method Detail


    - #### stratify

      ```
      MultiLabelInstances[] stratify(MultiLabelInstances data,
                                   int folds)
      ```

      Creates a number of folds via stratified sampling

      Parameters:
      :   `data` - a multi-label dataset
      :   `folds` - the number of folds to sample

      Returns:
      :   an array of multi-label datasets, one for each fold


- Overview
- Package
- Class
- Use
- Tree
- Deprecated
- Index
- Help

- Prev Class
- Next Class

- Frames
- No Frames

- All Classes

- Summary:
- Nested |
- Field |
- Constr |
- Method

- Detail:
- Field |
- Constr |
- Method
